# Metabolomics reveal how strong water binding naturally enhances wheat seed longevity and Fusarium resistance

**DOI:** 10.1007/s13659-025-00559-y

**Published:** 2026-01-09

**Authors:** Lorenzo Goglia, Vito Campanella, Agata Rascio

**Affiliations:** 1 Institute for Sustainable Plant Protection, National Research Council, Piazzale Enrico Fermi, 1, 80055 Portici, NA Italy; 2 Research Centre for Plant Protection and Certification, Council for Agricultural Research and Economics, V. le Regione Siciliana Sud-Est 8669, 90100 Palermo, Italy; 3 Research Centre for Cereal and Industrial Crops, Council for Agricultural Research and Economics (Retired), S.S. 673 Km 25, 200, 71122 Foggia, Italy

**Keywords:** Wheat, Seed, Aging, *Fusarium* resistance, Water binding, Metabolomics

## Abstract

Seed water content affects wheat quality, storage, and food safety, influencing viability and fungal contamination risks. Grain moisture is influenced by both genetic and environmental factors, with agronomic practices aiming to optimize it. However, no genotype has been specifically developed for enhanced seed water properties, as the underlying biochemical and genetic mechanisms remain unclear. Using a durum wheat mutant (WM) with higher water affinity of leaves, compared to its wild-type cultivar, Trinakria (WT), this study investigates the biophysical and biochemical mechanisms affecting seed performance. Genotypic characterization of unaged seeds includes differential scanning calorimetry, metabolomic profiling, and functional analyses of water uptake rate, dehydration rate, and seed coat electrical resistance. Germination and *Fusarium* resistance were examined, too in both unaged and aged seeds. As for the leaves, WM seeds exhibit higher water-binding strength than WT. Metabolomic analysis revealed a higher polar/apolar ratio in WM (83 vs. 72 in WT), with significantly greater myo-inositol and raffinose content and lower levels of unsaturated fatty acids. No differences in seed imbibition velocity or dehydration velocity were observed, but WM showed lower seed coat electrical resistance, indicating greater free water retention on the seed surface. Under low *Fusarium* inoculum concentrations or in the absence of pathogens, aged WM seeds showed higher germination rates and vigor than WT. As an inherited trait, selecting for strong water-binding capacity, increased osmoprotective compounds, and lower unsaturated acid content could contribute sustainably improve seed longevity and *Fusarium* resistance.

**Graphical Abstract:**

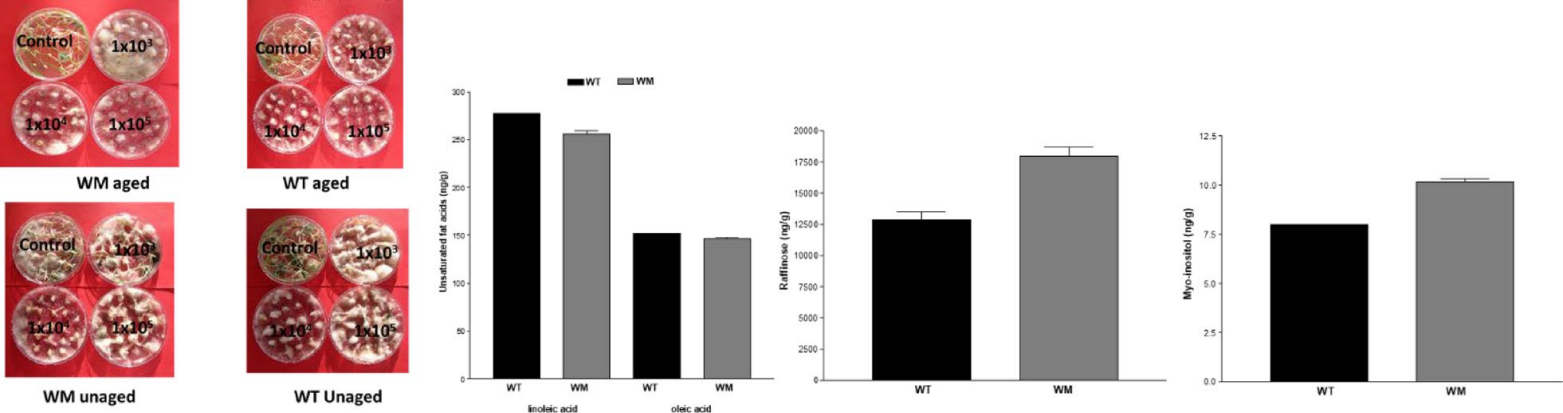

## Introduction

Seed moisture is commonly measured as an indicator of wheat seed quality, alongside other key traits such as protein content, hectoliter weight, 1000-seed weight, gluten content and yellow pigments. It influences harvesting efficiency, transportation costs, milling yield, shelf life, storage conditions, but also food safety.

In European Union, grain storage at m.c. up to 14.5% is recommended [1]. Seeds with higher water content are more susceptible to losing their viability: the ability to germinate and develop into a healthy plant under suitable conditions [2, 3]. This not only leads to yield loss but also increases the risk of fungal production of toxic substances, a major factor in non-compliance with food safety standards. For these reasons, the Italian law (no. 580 of 4 July 1967) prohibits the milling of grains spoiled due to excess humidity.

Based on the strength of tissue-water binding, different  types of water (bound water, weakly bound water, and free water) exist in almost all biological tissues [4, 5] and play essential roles in various physiological and pathological processes. During seed maturation, the gradual loss of free water leads to increased cytoplasmic concentration and viscosity until it solidifies into a glassy state, a process accompanied by cell shrinkage [6]. The movement of free water toward cell walls, driven by bound water interactions [7], imparts hyper elastic properties of very dry tissues [8, 9], may help seeds to withstand extreme dehydration modulating cell shrinkage [9] and then facilitating seed swelling during germination [10]. Some water-binding  compunds like trehalose, raffinose, and polyols facilitate the transition into a glassy state [11].

Grain moisture is influenced by both environmental and genetic factors [12]. Various agronomic and technological practices have been developed to optimize seed moisture for germination, harvesting, and storage. There is also potential for breeding, as genotypic differences in seed water status exist among cereal varieties [13]. To our knowledge, no genotype has ever been specifically selected for improved seed moisture or better water properties, as the underlying genes and mechanisms remain unidentified.

In this study, seeds from a durum wheat mutant with higher leaf water affinity, and its wild-type cultivar, Trinakria were analyzed using differential scanning calorimetry, metabolomic profiling, water rehydration and dehydration rate, and electrical resistance of the seed coat. Both properly stored (unaged) and poorly stored (aged) seeds were included to evaluate the functional effects of water status changes on germination and resistance to *Fusarium* infection.

Our findings provide deeper insights into the biophysical and biochemical determinants of seed performance and quality, which may serve as a basis for selecting  improved wheat genotypes.

## Material and methods

Seeds of WT and WM were collected from the fields of CREA-CI farm, located in Foggia (Italy) (41◦28 N, 15◦34 E; 76 m a.s.l.) in 2011 and in 2016 years. Samples collected in 2011(here named aged) were stored at room temperature until 2018. Samples collected in 2016 (referred to as unaged) were stored, at 4 °C and 40% RH, in the germplasm storage chambers of CREA-CI and analyzed in 2018.

### Analysis of unaged seeds

#### Differential enthalpy of water adsorption

The adsorption isotherm curves, at 5 and 20 °C were constructed, using a DVS (Dynamic Vapor Sorption) instrument (Q5000SA, TA instruments, New Castle, Delaware, USA). For both WT and WM seeds, approximately 3.7 mg of seeds were maintained at 40 °C for 4 h to reduce the water activity (aw) level, eliminate any surface-adsorbed vapor, and establish a baseline for dry mass. To generate the adsorption isotherms, the seeds were sequentially exposed to progressively increasing air relative humidity (RH) levels, ranging from 0 to 90%, with each step lasting 4 h.

The differential enthalpy of water adsorption (ΔH) was calculated according to the Clausius–Clapeyron equation [14]:

$$\Delta {\text{H}}\,\, = \,\,{\text{R x T}}^{{1}} {\text{T}}^{{2}} {\text{x ln}}({\text{aw}}^{{\text{l}}} /{\text{aw}}^{{2}} )$$

where AH is the differential enthalpy (in J/mol water) for a given water content, aw^l^ and aw^2^ were the relative vapor pressures at the lower (T^1^) and higher (T^2^) temperatures, respectively. R is the ideal gas constant (8.314 J/mol/°K), and T is temperature expressed in degrees Kelvin.

#### Metabolomic analysis

Unaged samples of WT and WM seeds were freeze dried, milled (Pulverisette^®^ 7 Planetary Micro Mill; Classic Line, Fritsch) with an agate jar and balls, and stored at 25 °C until analysis. The extraction, derivatisation and analysis of these samples for the profiling of the polar and non-polar metabolites were performed using a gas chromatography and mass spectrometry (GCMS) based approach, following protocols described previously [15]. All of the analyses were performed for three technical replicates, for each of two biological replicates. Briefly, 100 mg dry weight of each sample was extracted using a mixture of methanol (1 mL), ultrapure water (1 mL), and trichloromethane (3 mL), added sequentially. The samples were stored at 4 °C for 30 min and then centrifuged at 4000 g for 10 min at 4 °C. Aliquots (50 mL) of the polar and the trichloromethane phases (1 mL) were dried in a Speedvac for further analysis. The polar fraction was redissolved and derivatised for 90 min at 37 °C in methoxyamine hydrochloride in pyridine (70 mL; 20 mg/mL), followed by incubation with N-methyl-N-(trimethylsilyl) trifluoroacetamide (120 mL) at 37 °C for 30 min. The polar metabolites were analysed using GC (Agilent 6890N, Agilent Technologies, USA) coupled to quadrupole MS (Agilent 5973, Agilent Technologies, USA). Samples (1 mL) were injected in splitless mode, with GC separation on an HP-5 ms capillary column (60 m; 0.25 mm i.d.; 0.25 mm film thickness). Helium was used as the carrier gas, at a constant flow rate of 1 mL/min. For the analysis of polar metabolites, the injection temperature, transfer line, and ion source were set at 280 °C, and the quadrupole was adjusted to 180 °C. The oven was kept at 70 °C for 1 min, then increased at a rate of 5 °C/min to 310 °C, where it was held for 15 min. Subsequently, the temperature was increased to 340 °C, and held for 1 min. The spectrometer was operated in electron-impact mode at 70 eV, the scan range was from 30 to 700 amu, and the mass spectra were recorded at 2.21 scan/s. The non-polar metabolites were analysed as above, with minor modifications: the injection temperature and the transfer line were set at 250 °C; the oven was kept at 70 °C for 5 min, then increased at a rate of 5 °C/min to 310 °C, and held for 1 min. Subsequently, the system was equilibrated for 6 min at 70 °C before sample injection. The mass spectra were recorded at 2.28 scans/s, and the scan range was from 50 to 700 amu. The metabolites were identified through comparisons of the MS data with those of 2008 database of the National Institute of Standards and Technology (NIST) and with a custom library obtained with reference compounds. The GC–MS quantification was performed using the Chemstation software, by comparison with standard calibration curves obtained in the range of 0.04 ng-2.00 ng, The standards and all of the chemicals used were of HPLC grade and were from Sigma-Aldrich Chemical Co. (Deisenhofen, Germany). The N-methyl-N-(trimethylsilyl) trifluoroacetamide was from Fluka.

#### Seed imbibition rate and dehydration rate

The water imbibition rate at increasing time intervals was determined using 5 WT and 5 WM seeds from lots collected in the same field and cropping year. Each seed was put in 50 ml of distilled water in the light to determine the weight increase and the electrical resistance at 4 h and 24 h intervals. After then, to examine the dehydration kinetic at 35 °C, the seeds were weighted at 10 min intervals during the following 60 min. Electrical resistance (SER) was estimated as previously reported [16], using a digital sensor (Profi + ; Powerfix) whose scale values are inversely functions of the electrical resistance applied to the electrodes. Water uptake rate was calculated in relation to seed mass at time = 0. Percentage water uptake was calculated following [10] in relation to seed mass at t0 as follows:

$$\% {\text{Ws}}\,\,\, = \,\,\,\left[ {\left( {{\text{Wi}}{ - }{\text{Wd}}} \right) / {\text{Wd}}} \right] \, \cdot{ 1}00$$

where Ws = increase in mass of seed, Wi = mass of seed after a given interval of imbibition, and Wd = seed mass at t0.

### Analysis of unaged and aged seeds

#### Viability and resistance to Fusarium culmorum

Seed resistance to *Fusarium* and seed viability [17] were analysed using two sample types:

1. Seed collected in 2011 and stored at room temperature for 5 years (aged);
2. Seed collected in 2015 and stored at a temperature of 4 °C and a relative humidity of 40% for 2 years (unaged).

All seeds were surface-sterilized by stirring in a 1% hypochlorite solution for 15 min, then rinsed five to six times with sterile deionized water and allowed to air dry at room temperature. Subsequently they were inoculated by immersion (3 min) in a conidial suspension of *Fusarium culmorum* (is.162). The isolate of *F*. *culmorum* was grown on Petri plates containing 12 ml of PDA (39 g/l; Oxoid Ltd. Basingstoke, UK) incubated at 20 ± 2 °C under near-ultraviolet light alternating light/darkness on 12-h cycles. 15-day-olf cultures were flooded with 10 ml of sterile distilled water and then conidia were dislodged with a sterile bacteriology loop by scraping the surface of the mycelium. After filtering through sterile gauze to remove mycelial fragments, the concentration of conidia was measured using a Thoma counting chamber (HGB Henneberg-Sander GmbH, Lutzellinder, Germany). The resulting conidia concentration was diluted to 1 × 10^3^, 1 × 10^4^, and 1 × 10^5^ CFU/ml with sterile distilled water. As a control, seeds were immersed in sterile distilled water. The seeds were then placed in Petri dishes containing a potato dextrose agar (PDA) substrate, and the dishes were incubated in the dark at 20 °C. Two replicates of 20 seeds each were used for each concentration.

After 7 days, the following parameters were the germination rate together with the shoot and root development of 10 plants per treatment were measured as previously reported [18]. The data obtained were subjected to analysis of variance, and the means were separated using Duncan’s test (*p* ≤ *0.01*).

Seedling vigor index (SVI), was calculated according to the formula of [15]:

$$\left( {{\mathbf{S}}\, + \,{\mathbf{R}}} \right) \, {\mathbf{x}} \, {\mathbf{G}}/{\mathbf{100}}$$

where:

- **S** = Vegetative development of the shoot,
- **R** = Vegetative development of the roots,
- **G** = Germination percentage.

## Results

### Moisture isotherms

**Fig. 1:**
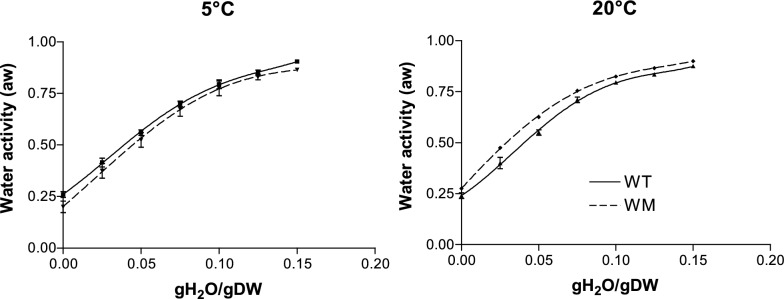
Water adsorption isotherms of WT and WM at 5 and 20 °C. Means ± SE

**Fig. 2:**
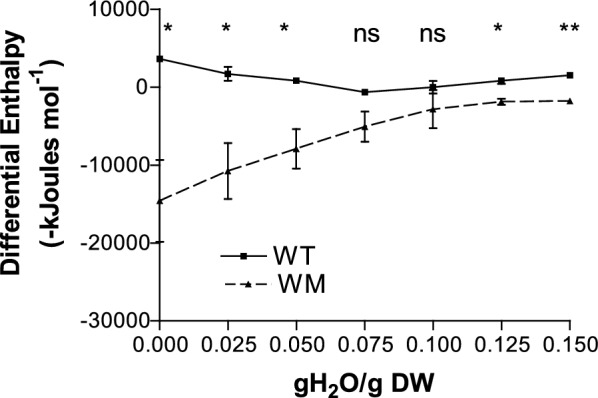
Differential energies of water adsorption of WT and WM seeds at different moisture contents. * significant at 0.05 probability level, ** significant at 0.01 probability level, *ns* not significant at 0.05 probability level, on the basis of Student’s test

The water adsorption isotherms of both genotypes exhibit a curvilinear pattern, reflecting the presence of water fractions with varying affinities to the seed tissues (Fig. 1). The hydration enthalpy (Fig. 2) of WM was significantly more negative than that of WT, particularly at moisture levels below 0.075 gH2O/gDW, indicating a stronger water-seed binding force.

### Seed chemical composition

Metabolomic analysis revealed an appreciable content for 27 out of the 32 analyzed compounds (Table 1), with a polar/ apolar ratio equal to 83 and 72 for WM and WT, respectively. Sucrose, raffinose and maltose were the most abundant polar compound, linoleic and oleic those apolar. As compared to the WT, WM has significant greater myo-inositol and raffinose content while, the unsaturated cis-9-octadecenoic acid (oleic acid) and cis,cis-9,12-octadecadienoic acid (linoleic acid) were significantly lower.

**Table 1:** Results of metabolomic analysis and student’s paired t-test

		WT	WM		
		ng/g	ng/g	t	p
1	Aspartic acid 3 TMS	4,3	4,3	0,00	1,000
2	Glutamic acid	14,7	17,1	−0,91	0,461
3	Isocitric acid	0,0	3,0	−1,00	0,423
4	Citric acid	0,0	3,3	−1,00	0,423
5	Fructose	197,8	234,9	−3,70	0,066
6	Galactose	47,7	51,1	−0,66	0,575
7	Glucose + mannosio	123,7	131,7	−0,52	0,657
8	Mannitol	14,2	15,9	−1,23	0,345
9	myo-inositol	**8,0**	**10,2**	−**13,00**	**0,006**
10	Triptophane 3 TMS	28,8	67,2	−1,17	0,362
11	Sucrose	69199,1	79213,6	−0,95	0,441
12	Maltose + turanose	4472,5	4424,4	0,06	0,956
13	Raffinose	**12860,2**	**17936,2**	−**5,18**	**0,035**
14	Oxalic acid	202,2	143,5	1,77	0,219
15	Glycerol	27,3	36,5	−2,58	0,123
16	Phosphate	45,2	38,4	2,83	0,106
17	Malic acid	72,6	87,0	−1,08	0,394
18	Hexadecanoic acid	153,7	138,1	2,82	0,106
19	cis,cis-9,12-octadecadienoic (linoleic)	**277,7**	**255,5**	**5,75**	**0,029**
20	cis-9-octadecenoic acid (oleic)	**151,8**	**146,1**	**4,36**	**0,049**
21	Octadecanoic acid	124,0	122,3	2,02	0,181
22	Tricosane	56,5	54,9	1,30	0,323
23	1,3-dihydroxy-5-nonadecyl-benzene	27,5	24,7	1,60	0,251
24	1,3-dihydroxy-5-heneicosyl-benzene	139,5	124,0	2,39	0,139
25	Campsterol	131,0	228,3	−0,74	0,536
26	b-sitosterol	70,8	64,3	2,84	0,105
27	1,3-dihydroxy-5-tricosyl-benzene	79,0	76,6	2,20	0,159

Bold text was used to highlight the parameters that are significantly different

### Imbibition rate and dehydration rate

**Fig. 3:**
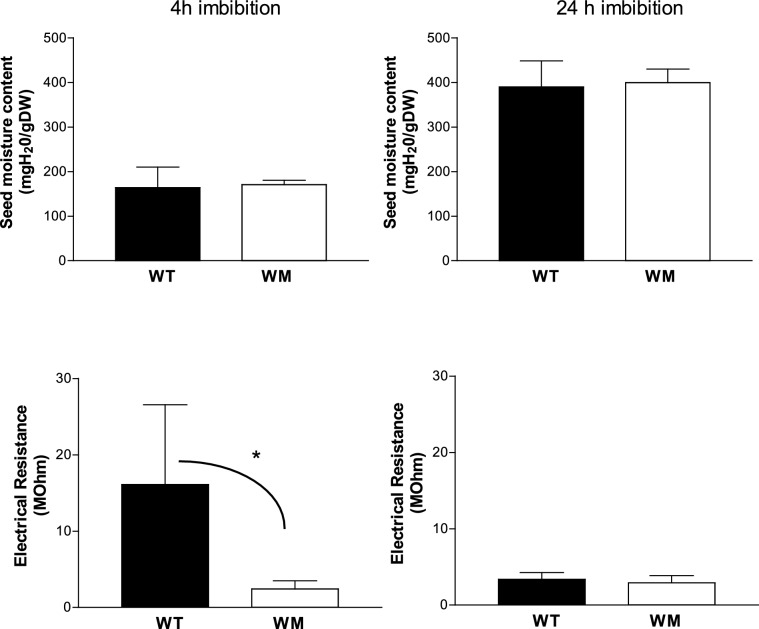
The seed water content and the coat electrical resistance determined after 4 h and 24 h imbibition. * significant at 0.05 probability level

Figure 3 shows the water content for unit of dry matter and the electrical resistance determined after 4 h  (on the left) and 24 h imbibition (on the right). After 4 h, the WM had the same water content but lower electrical resistance of the seed surface than WT; after 24 h of imbibition, the two genotypes did not differed for both traits.

**Fig. 4:**
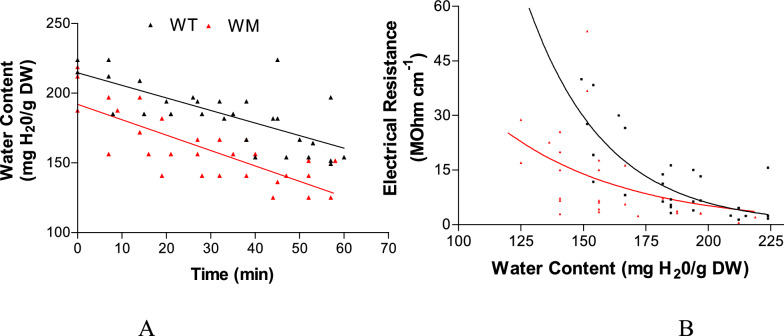
**A** Dehydration kinetics and **B** electrical resistance of the uppermost layer as seed water content decreases

Examining the rate of water loss in WM and WT, both exhibited the same dehydration velocity (Fig. 4A). However, the electrical resistance of the uppermost seed layers was lower in WM, especially at the lowest moisture content (Fig. 4B).

### Seed viability and Fusarium susceptibility

**Fig. 5:**
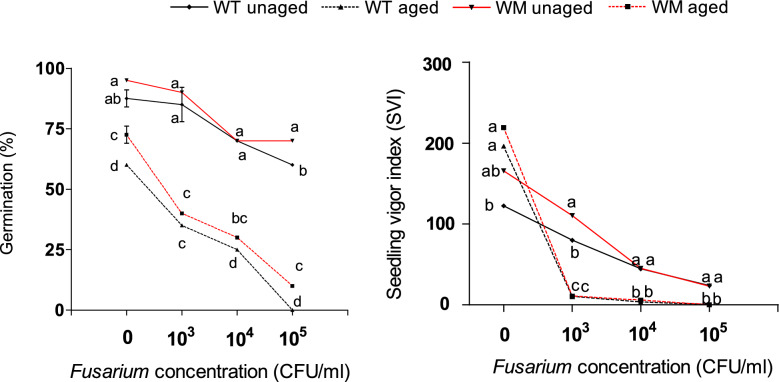
Germination and seedling vigor of aged and unaged seeds at increasing *Fusarium* inoculation concentrations. At each *Fusarium* concentration, means with the same letters are not different according to Fisher’s least significant difference

Aging causes a significant reduction in germination for both the genotypes (Fig. 5), while seed vigor was higher for uninfected, aged seed of both genotypes. On the average of the two treatments the mutant exhibits better germination (5% higher) than the WT, and this advantage was above all significant for aged seeds or those infected by *Fusarium*. Analogously seed vigor of WM was on average higher than WT in the absence of pathogens or at low inoculum concentrations (10^3^). At higher concentrations (> 10^3^) the seeds of both genotypes exhibited similar decreasing vigor with increasing *Fusarium* concentration. Interestingly, in the absence of pathogens, unaged WT seeds displayed lower vigor than aged seeds.

## Discussion

Alongside agronomic advancements, genetic strategies are the most promising approach for enhancing seed quality [19]. Emerging technologies have the potential to accelerate breeding efforts [20], however, a deeper understanding of the genes and physiological mechanisms that enhance seed performance is essential.

Consistent with observations in the leaves [21], WM seeds exhibit higher water-binding strength than WT. The more negative hydration enthalpy of WM did not significantly affect seed imbibition or dehydration velocity. WM had the same water content but lower electrical resistance than WT in the uppermost seed layers, particularly at the lowest moisture content. Since electrical resistance is inversely proportional to water content, this suggests a different water distribution within the seed tissues, with WM retaining more free water on the seed coat.

Uninfected aged seeds exhibited lower germination but higher vigor than unaged seeds. This may be due to reduced free water availability and older microbial populations being less capable of hindering seedling development. Additionally, field conditions may have promoted the colonization of seeds by auxin-producing antagonistic bacteria, which stimulate seedling growth [22].

Minor genotypic differences were observed in the germination of unaged seeds, while aged WM seeds showed significantly higher germination rates and vigor than WT, especially in pathogen-free conditions or under low inoculum levels. This supports the observation that a high water-binding capacity is above all associated with reduced viability loss during seed storage [23].

Results of metabolomic analysis suggested the biochemical mechanisms contributing to the superior performance of WM. Its seeds contained higher levels of raffinose and myo-inositol, along with lower concentrations of unsaturated fatty acids. The first two are oligosaccharides of raffinose family (RFOs) known to play a key role in the resumption of respiration and seed-to-seedling transition, and to act as osmoprotectants that enhance seed resistance to *Fusarium* attacks [24]. Also the lower content of oleic and linoleic acids in WM may have contributed to improved seed longevity [25], considering that low content of unsaturated fatty acids decrease susceptibility to oxidative degradation and reduces the availability of substrates for fungal metabolism [26].

### Concluding remarks and future outlook

High seed water affinity proves to be a valuable wheat quality trait. Since the number and types of macromolecules primarily influence tissue water-binding strength, breeding, omics-guided selection, and metabolic engineering should prioritize enhancing osmoprotective compounds while reducing unsaturated acid content. The wheat mutant, selected for its high water-binding strength, represents a valuable source of alleles for sustainable breeding strategies to enhance seed longevity and pathogen resistance.

## Data Availability

The seeds of genotypes used in the manuscript are available upon request.
